# The Roles of a Secondary Data Analytics Tool and Experience in Scientific Hypothesis Generation in Clinical Research: Protocol for a Mixed Methods Study

**DOI:** 10.2196/39414

**Published:** 2022-07-18

**Authors:** Xia Jing, Vimla L Patel, James J Cimino, Jay H Shubrook, Yuchun Zhou, Chang Liu, Sonsoles De Lacalle

**Affiliations:** 1 Department of Public Health Sciences College of Behavioral, Social, and Health Sciences Clemson University Clemson, SC United States; 2 Cognitive Studies in Medicine and Public Health The New York Academy of Medicine New York City, NY United States; 3 Informatics Institute School of Medicine University of Alabama, Birmingham Birmingham, AL United States; 4 College of Osteopathic Medicine Touro University Vallejo, CA United States; 5 Patton College of Education Ohio University Athens, OH United States; 6 Russ College of Engineering and Technology Ohio University Athens, OH United States; 7 College of Art and Science California State University Channel Islands Camarillo, CA United States

**Keywords:** clinical research, observational study, scientific hypothesis generation, secondary data analytics tool, think-aloud method

## Abstract

**Background:**

Scientific hypothesis generation is a critical step in scientific research that determines the direction and impact of any investigation. Despite its vital role, we have limited knowledge of the process itself, thus hindering our ability to address some critical questions.

**Objective:**

This study aims to answer the following questions: To what extent can secondary data analytics tools facilitate the generation of scientific hypotheses during clinical research? Are the processes similar in developing clinical diagnoses during clinical practice and developing scientific hypotheses for clinical research projects? Furthermore, this study explores the process of scientific hypothesis generation in the context of clinical research. It was designed to compare the role of VIADS, a visual interactive analysis tool for filtering and summarizing large data sets coded with hierarchical terminologies, and the experience levels of study participants during the scientific hypothesis generation process.

**Methods:**

This manuscript introduces a study design. Experienced and inexperienced clinical researchers are being recruited since July 2021 to take part in this 2×2 factorial study, in which all participants use the same data sets during scientific hypothesis–generation sessions and follow predetermined scripts. The clinical researchers are separated into experienced or inexperienced groups based on predetermined criteria and are then randomly assigned into groups that use and do not use VIADS via block randomization. The study sessions, screen activities, and audio recordings of participants are captured. Participants use the think-aloud protocol during the study sessions. After each study session, every participant is given a follow-up survey, with participants using VIADS completing an additional modified System Usability Scale survey. A panel of clinical research experts will assess the scientific hypotheses generated by participants based on predeveloped metrics. All data will be anonymized, transcribed, aggregated, and analyzed.

**Results:**

Data collection for this study began in July 2021. Recruitment uses a brief online survey. The preliminary results showed that study participants can generate a few to over a dozen scientific hypotheses during a 2-hour study session, regardless of whether they used VIADS or other analytics tools. A metric to more accurately, comprehensively, and consistently assess scientific hypotheses within a clinical research context has been developed.

**Conclusions:**

The scientific hypothesis–generation process is an advanced cognitive activity and a complex process. Our results so far show that clinical researchers can quickly generate initial scientific hypotheses based on data sets and prior experience. However, refining these scientific hypotheses is a much more time-consuming activity. To uncover the fundamental mechanisms underlying the generation of scientific hypotheses, we need breakthroughs that can capture thinking processes more precisely.

**International Registered Report Identifier (IRRID):**

DERR1-10.2196/39414

## Introduction

A hypothesis is an educated guess or statement about the relationship between 2 or more variables [1,2]. Scientific hypothesis generation is a critical step in scientific research that determines the direction and impact of research investigations. However, despite its vital role, we do not know the answers to some basic questions about the generation process. Some examples are as follows: “Can secondary data analytics tools facilitate the process?” and “Is the scientific hypothesis generation process for clinical research questions similar to differential diagnosis questions?” Traditionally, the scientific method involves delineating a research question and generating a scientific hypothesis. After formulating a scientific hypothesis, researchers design studies to test the scientific hypothesis to determine the answers to research questions [1,3].

Scientific hypothesis generation and scientific hypothesis testing are distinct processes [1,4]. In clinical research, research questions are often delineated without the support of systematic data analysis and are not data driven [1,5,6]. Using and analyzing existing data to facilitate scientific hypothesis generation is considered ecological research [7,8]. An ever-increasing amount of electronic health care data is becoming available, much of which is coded. These data can be a rich source for secondary data analysis, accelerating scientific discoveries [9]. Thus, many researchers have been exploring data-driven scientific hypothesis generation guided by secondary data analysis [1,10]. This includes various fields, including genomics [4]. However, exactly how a scientific hypothesis is generated, even as shown by secondary data analysis in clinical research, is unknown. Understanding the detailed process of scientific hypothesis generation could improve the efficiency of delineating clinical research questions and, consequently, clinical research. Therefore, this study investigates the process of formulating scientific hypotheses guided by secondary data analysis. Using these results as a baseline, we plan to explore ways of supporting and improving the scientific hypothesis–generation process and to study the process of formulating research questions as long-term goals.

Electronic health record systems and related technologies have been widely adopted in both office-based physician practices (86% in 2019) [11] and hospitals (overall 86% in 2022), and types vary based on hospital types [12] across the United States. Thus, vast amounts of electronic data are continuously captured and available for analysis to guide future decisions, uncover new patterns, or identify new paradigms in medicine. Much of the data is coded using hierarchical terminologies, and some of these commonly used terminologies include the International Classification of Diseases, 9th Revision-Clinical Modification (ICD9-CM) [13] and 10th Revision-Clinical Modification (ICD10-CM) [14], Systematized Nomenclature of Medicine-Clinical Terms (SNOMED-CT) [15], Logical Observation Identifiers Names and Codes (LOINC) [16], RxNorm [17], Gene Ontology [18], and Medical Subject Headings (MeSH) [19]. We used the coded data sets by hierarchical terminologies *as examples* of existing data sets to facilitate and articulate the scientific hypothesis–generation process in clinical research, especially when guided by secondary data analyses. Algorithms [20,21] and a web-based secondary data analytics tool [22-25] were developed to use the coded electronic data (ICD9 or MeSH) in order to conduct population studies and other clinically relevant studies.

Arocha et al and Patel et al [26,27] studied the directionality of reasoning in scientific hypothesis–generation processes and evaluation strategies (of confirmation or disconfirmation) for solving a cardiovascular diagnostic problem by medical students (novice) and medical residents (experienced). The reasoning directions include forward (from evidence to a scientific hypothesis) and backward (from a scientific hypothesis to evidence). More experienced clinicians used their own underlying situational knowledge about the clinical condition, while the novices used the surface structure of the patient information during the diagnosis generation process. The studies by Patel et al [28,29] and Kushniruk et al [30] used inexperienced and experienced clinicians with different roles, levels of medical expertise, and corresponding strategies to diagnose an endocrine disorder. In these studies, expert physicians used more efficient strategies (integrating patient history and experts’ prior knowledge) to make diagnostic decisions [28,30,31]. All these studies focused on hypothesis generation in solving diagnostic problems. Their results set the groundwork for reasoning in the medical diagnostic process. Their findings regarding the generation of diagnostic hypotheses by experienced and inexperienced clinicians via different processes helped us formulate and narrow our research questions. Their methodology involved performing predefined tasks, recording “think-aloud” sessions, and transcribing and analyzing the study sessions. Making a diagnosis is a critical component of medicine and a routine task for physicians. In contrast, generating scientific hypotheses in clinical research focuses on establishing a scientific hypothesis or doing further searches to explore alternative scientific hypotheses for research. The difference between the 2 can be demonstrated by 2 enterprises. In clinical practice, the goal of generating a diagnostic hypothesis is to make decisions about patient care and the task is time constrained, while in scientific research, time is not similarly constrained and the task is to explore various scientific hypotheses to formulate and refine the final research question. In both making a medical diagnosis in clinical practice and scientific thinking, generating initial hypotheses depends on prior knowledge and experience [32,33]. However, in scientific thinking, analogies and associations play significant roles, in addition to prior knowledge, experience, and reasoning capability. Analogies are widely recognized as playing vital heuristic roles as aids to discovery [32,34], and these have been employed in a wide variety of settings and have had considerable success in generating insights and formulating possible solutions to existing problems.

This makes it essential to understand the scientific hypothesis–generation process in clinical research and to compare this process with generating clinical diagnoses, including the role of experience during the scientific hypothesis–generation process.

This study explores the scientific hypothesis–generation process in clinical research. It investigates whether a secondary data analytics tool and clinician experience influence the scientific hypothesis–generation process. We propose to use direct observations, think-aloud methods with video capture, follow-up inquiries and interview questions, and surveys to capture the participants’ perceptions of the scientific hypothesis–generation process and associated factors. The qualitative data generated will be transcribed, analyzed, and quantified.

We aim to test the following study hypotheses:

1. Experienced and inexperienced clinical researchers will differ in generating scientific hypotheses guided by secondary data analysis.
2. Clinical researchers will generate different scientific hypotheses with and without using a secondary data analytics tool.
3. Researchers’ levels of experience and use of secondary data analytics tools will interact in their scientific hypothesis–generation process.

In this paper, we used the term “research hypothesis” to refer to a statement generated by our research participants, the term “study hypothesis” to refer to the subject of our research study, and the term “scientific hypothesis” to refer to the general term “hypothesis” in research contexts.

## Methods

### Design

This manuscript introduces a study design and uses a mixed methods approach. The study includes assessment of direct observational, utility, and usability study designs. Surveys, interviews, semipredefined tasks, and capturing screen activities are also utilized. The modified Delphi method is also used in the study.

### Ethics Approval

The study has been approved by the Institutional Review Board (IRB) of Clemson University, South Carolina (IRB2020-056).

### Participants and Recruitment

Experienced and inexperienced clinical researchers, and a panel of clinical research experts, have been recruited for this study. The primary criterion used to distinguish subjects in the 3 groups is the level of their experience in clinical research. Table 1 summarizes the requirements for clinical researchers, expert panel members, and the computers that clinical researchers use during the study sessions. Participants are compensated for their time according to professional organization guidelines.

**Table 1:** Summary of the criteria for study participants and clinical research expert panel members.

Variable	Inexperienced clinical researchers^a^	Experienced clinical researchers^a^	A panel of clinical research experts
Participation in research hypothesis generation and study design	≤2 years	Leading role ≥5 and <10 years	Leading role ≥10 years
Participation in data analysis of study results	≤2 years	Leading role ≥5 and <10 years	Leading role ≥10 years
Publications in clinical research	Not required	≥5 as a leading author, including first, correspondence, or senior author for original studies	≥10 as a leading author, including at least one article in a high-impact journal in the past 5 years
Review experience in clinical research for conferences, journals, or grants	Not required	Not required	≥10 years
Internet connection	Required	Required	Required
Microphone	Required	Required	Not required
Software package for data analysis (eg, Microsoft Excel and R)	Required	Required	Not required
Tools to facilitate research hypothesis generation, if available	Required	Required	Not required

^a^If a participant has clinical research experience between 2 and 5 years, the decisive factor for the experienced group will be 5 publications for original studies as the leading author.

To recruit participants, invitational emails and flyers have been sent to collaborators, along with other communication means, including mailing lists, such as those of working groups of the American Medical Informatics Association, South Carolina Clinical & Translational Research Institute newsletters, and PRISMA health research newsletters, and Slack channels, such as National COVID Cohort Collaborative communities. All study sessions are conducted remotely via video conference software (Webex, Cisco) and recorded via a commercial software (BB FlashBack, Blueberry Software).

### Introduction to VIADS

**Figure 1:**
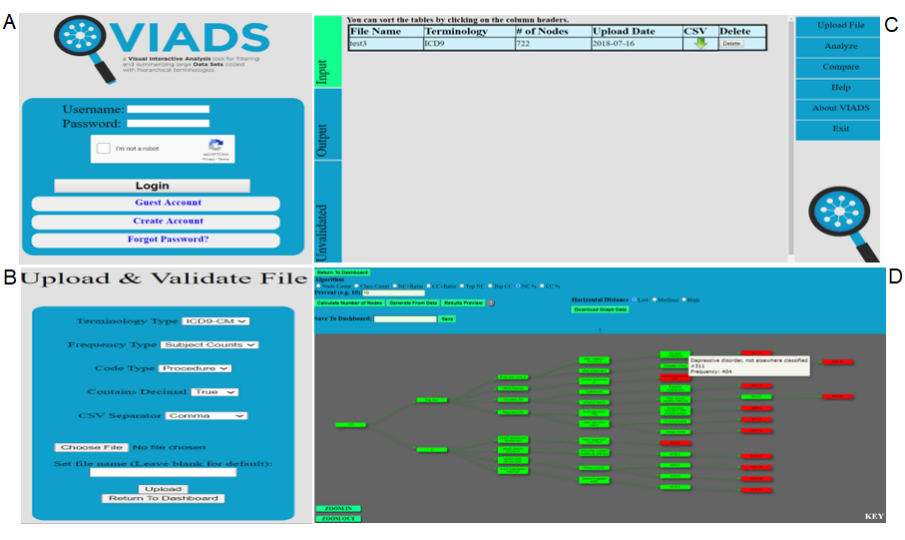
Selected screenshots of VIADS. (A) Homepage; (B) validation module; (C) dashboard; (D) a graph coded using International Classification of Diseases, 9th Revision-Clinical Modification (ICD9-CM) codes and generated by VIADS.

In this study, we have used VIADS as an example of a secondary data analytics tool. VIADS is a visual interactive analytical tool for filtering and summarizing large health data sets coded with hierarchical terminologies [22,23,35]. It is a cost-free web-based tool available for research and educational purposes. VIADS can be used by both registered users and guest users without registration. VIADS and the underlying algorithms were developed previously by the authors [20,21,24]. VIADS was designed to use codes and usage frequencies from terminologies with hierarchical structures to achieve the following objectives: (1) provide summary visualizations, such as graphs, of data sets; (2) filter data sets to ensure manageable sizes based on user selection of algorithms and thresholds; (3) compare similar data sets and highlight the differences; and (4) provide interactive, customizable, and downloadable features for the graphs generated from the data sets. VIADS is a useful secondary data analytics tool that can facilitate decision-making by medical administrators, clinicians, and clinical researchers. For example, VIADS can be used to track longitudinal data of a hospital over time and can explore trends and detect diagnosis trends and differences over time. VIADS can also be used to compare 2 similar medications and the medical events associated with the medications in order to provide detailed evidence to guide more precise clinical use of the medications [20]. This study provides evidence of the different information needs of physicians and nurses via the algorithms of VIADS [21]. Figure 1 shows example screenshots of VIADS.

Meanwhile, we recognize that VIADS can only accept coded clinical data and their associated use frequencies. Currently, VIADS can accept data sets coded using ICD9-CM, ICD10-CM, and MeSH. This can limit the types of scientific hypotheses generated by VIADS.

### Preparation of Test Data Sets

We have prepared and used the same data sets for this study across different groups in order to reduce the potential biases introduced by different data sets. However, all data sets (ie, input files) used in VIADS are generally prepared by users within specific institutions. Table 2 summarizes the final format of data sets and the minimum acceptable sizes of data sets needed for analysis in VIADS. The current version of VIADS is designed to accept all data sets coded by the 3 types of terminologies (ICD9-CM, ICD10-CM, and MeSH) listed in Table 2. No identified patient information is included in the data sets used in VIADS, as the data sets contain only the node identification (ie, terminology code) and usage frequencies.

**Table 2:** Acceptable formats and data set sizes in VIADS.

Data set^a^ and graph node ID (code)		Usage frequency
**ICD9-CM^b^ data set**		
	300.00	2223
	278.00	5567
	… …^c^	…
**ICD10-CM^d^ data set**		
	O10.01	5590
	E11.9	50,000
	… …^c^	…
**MeSH^e^ data set**		
	A0087342	16,460
	A0021563	4459
	… …^c^	…

^a^Acceptable data set sizes for Web VIADS are as follows: patient counts ≥100 and event counts ≥1000.

^b^ICD9-CM: International Classification of Diseases, 9th Revision-Clinical Modification.

^c^There are many more codes in addition to the 2 examples provided.

^d^ICD10-CM: International Classification of Diseases, 10th Revision-Clinical Modification.

^e^MeSH: Medical Subject Headings.

The usage frequencies of the data sets used in VIADS can be either of the following: patient counts (the number of patients associated with specific ICD codes in the selected database) or event counts (the number of events [ICD codes or MeSH terms] in the selected database).

An ancestor-descendant table, which contains 1 row for each node and each of its distinct descendants, can calculate class counts easier and more accurately without counting the same node multiple times. These implementation details have been discussed in greater detail in prior publications [20,21].

A publicly accessible data source [36], including ICD9-CM codes, has been used to generate the needed input data sets. The patient counts are used as frequencies. Although ICD10-CM codes are now used in the United States, ICD9-CM data spanning the past several decades are available in most institutions across the country. Therefore, ICD9-CM codes have been used to obtain historical and longitudinal perspectives.

### Instrument Development

**Figure 2:**
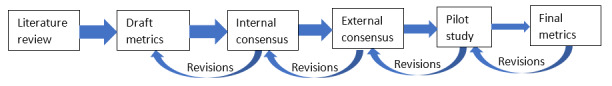
Development process for metrics to evaluate research hypotheses in clinical research.

Metrics have been developed to assess research hypotheses generated during the study sessions. The development process goes through iterative stages (Figure 2) via Qualtrics surveys, emails, and phone calls. First, a literature review is conducted to outline draft metrics. Then, the draft metrics are discussed and iteratively revised until all concerns are addressed. Next, the revised metrics are distributed to the entire research team for feedback. The internal consensus processes follow a modified Delphi method [37]. Modifications at this point primarily include transparent and open discussions conducted via email among the research team and anonymous survey responses received before and after discussions. The main difference between our modification and the traditional Delphi method is the transparent discussion among the whole team via emails between the rounds of surveys.

The performance of scientific hypothesis–generation tasks will be measured using metrics that include the following qualitative and quantitative measures: validity, significance, clinical relevance, feasibility, clarity, testability, ethicality, number of total scientific hypotheses, and average time used to generate 1 scientific hypothesis. In an online survey, the panel of clinical research experts will assess the generated research hypotheses based on the metrics we have developed.

**Figure 3:**
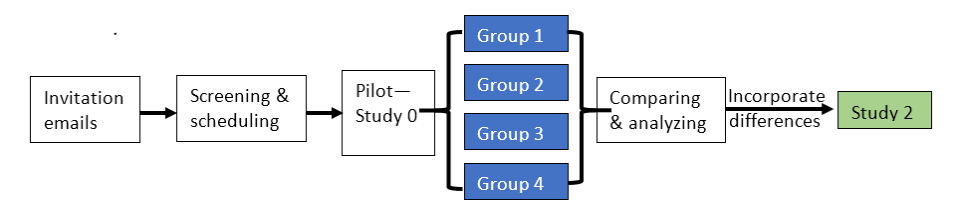
Summary of the study procedures. Blue boxes indicate data collected in Study 1.

A survey (Multimedia Appendix 1) is administered for the first 4 groups at the end of the research hypothesis–generation study sessions (Figure 3). The groups that use VIADS also complete a modified System Usability Scale (SUS) questionnaire (Multimedia Appendix 2) evaluating the usability and utility of VIADS. The follow-up or inquiry questions and parameters generated during the think-aloud process used in Study 1 (Figure 3) are as follows:

- What are the needed but currently unavailable attributes that would help clinical researchers generate research hypotheses? The question is similar to a wish list of features facilitating research hypothesis generation.
- Follow-up questions clarify potential confusion during the think-aloud processes or enable meaningful inquiries when unexpected or novel questions emerge during observations [38]. However, these questions have been kept to a minimal level to avoid interrupting clinical researchers’ thinking processes. This method complements the data from the think-aloud video captures.
- Can the list of items in Multimedia Appendix 3, which has been compiled from traditional clinical research textbooks [1-3,8,39] on scientific hypothesis generation and research question formulation, facilitate clinical researchers’ research hypothesis generation when guided by secondary data analysis?

A survey will be developed based on the comparative results of the first 4 groups and administered at the end of Study 2. The identified differences from Study 1 will be the focus of the survey to determine whether these differences are helpful in Study 2.

### Study Design

Study 1 tests all 3 study hypotheses. If the study hypotheses are supported in Study 1, we will conduct a follow-up study (Study 2) to examine whether and how the efficiency or quality of research hypothesis generation may be improved or refined.

We will use the think-aloud method to conduct the following tasks: (1) observe the research hypothesis–generation process; (2) transcribe data, and analyze and assess if VIADS [22] and different levels of experience of clinical researchers influence the process; and (3) assess the interactions between VIADS (as an example of a secondary analytical tool) and the experience levels of the participants during the process. Figure 3 summarizes the Study 1 and Study 2 procedures.

#### Study 1

Experienced and inexperienced clinical researchers conduct research hypothesis–generation tasks using the same secondary data sets. The tasks are captured to describe participants’ research hypothesis–generation processes when guided by analysis of the same data sets. VIADS is also used as an example of a secondary data analytics tool to assess participants’ research hypothesis–generation processes. Accordingly, 2 control groups do not use VIADS, while 2 intervention groups do use it.

A pilot study, Study 0, was conducted in July and August 2021 for each group to assess the feasibility of using the task flow, data sets, screen capture, audio and video recordings, and study scripts. In Study 1, 4 groups are utilized. Table 3 summarizes the study design and participants in each group. The 4 groups will be compared in order to detect the primary effects of the 2 factors and their interactions after completing Study 1. After recruitment, the participants are separated into experienced or inexperienced groups based on predetermined criteria. Then, the participants are randomly assigned to a group that uses VIADS (3-hour session, with 1 hour to conduct VIADS training) or does not use VIADS (2-hour session) via block randomization.

**Table 3:** Design of Study 1 for assessment of the hypothesis–generation process in clinical research.

Variable	Number of experienced clinical researchers	Number of inexperienced clinical researchers
Not using VIADS	8 (Group 1)	8 (Group 2)
Using VIADS	8 (Group 3)	8 (Group 4)

We conduct 1 study session individually with each participant. The participants are given the same data sets from which to generate research hypotheses with or without VIADS during each study session. The same researcher observes the entire process and captures the process via think-aloud video recordings. Follow-up or inquiry questions from the observing researcher are used complementarily during each study session. The study sessions are conducted remotely via Webex. All screen activities are captured and recorded via BB FlashBack [40].

#### Study 2

If the results of Study 1 indicate group differences as expected (in particular, differences are found between experienced and inexperienced clinical researchers, along with differences between groups using VIADS and not using VIADS), Study 2 will be conducted to examine whether the efficiency of the research hypothesis–generation process could be improved. Specifically, we will analyze for group differences, identify anything related to VIADS, and incorporate them into VIADS. Then, we will test whether the revised and presumably improved VIADS increases the efficiency or quality of the research hypothesis–generation process. In this process, the group that has the lowest performance in Study 1 will be invited to use the revised VIADS to conduct research hypothesis–generation tasks again with the same data sets. However, at least 8 months will be allowed to pass in order to provide an adequate wash-out period. This group’s performance will be compared with that in Study 1.

If no significant difference can be detected between the groups using VIADS and not using VIADS in Study 1, we will use the usability and utility survey results to revise VIADS accordingly without conducting Study 2. If no significant difference can be detected between experienced and inexperienced clinical researchers, Study 2 will recruit both experienced and inexperienced clinical researchers as study participants. In this case, Study 2 will focus only on whether a revised version of VIADS impacts the research hypothesis–generation process and outcomes.

### Data and Statistical Analysis

While conducting the given tasks, the qualitative data collected via the think-aloud method will be transcribed, coded, and analyzed according to the grounded theory [41,42], a classical qualitative data analysis method. This data analysis has not begun yet because data are still being collected. Combined analysis of discourse [30], video recordings of the study sessions, and screen activities will be conducted. The main components or patterns that we will focus on during analysis include potential nonverbal steps, sequential ordering among different components (such as prioritization of the use of either experience or data) across groups, seeking and processing evidence, analyzing data, generating inferences, making connections, formulating a hypothesis, searching for information needed to generate research hypotheses, and so forth. Ideally, based on video analyses and observations, we plan to develop a framework for the scientific hypothesis–generation process in clinical research, which is guided by secondary data analytics. Similar frameworks exist in education and learning areas [43], but do not currently exist in the field of clinical research.

The outcome variable used is based on the participants’ performance in research hypothesis–generation tasks. The performance is measured by the quality (eg, significance and validity) and quantity of the research hypotheses generated through the tasks and the average time to generate 1 research hypothesis. At least three clinical research experts will assess each hypothesis using the scientific hypothesis assessment metrics that were developed for this study. The metrics include multiple items, each on a 5-point Likert scale. The details of the metrics are described in the Instrument Development section.

The data will be analyzed with a 2-tailed factorial analysis. We calculated the required sample size in G*Power 3.1.9.7 for a 2-way ANOVA. The sample size was 32 based on a confidence level of 95% (α=.05), effect size f=0.5, and power level of 0.8 (β=.20).

In Study 1, we will use descriptive statistics to report how many hypotheses were generated, average time spent per hypothesis, and how many hypotheses were evaluated for each participant. A 2-way ANOVA will be used to examine the main effects of VIADS and experience, as well as the interaction effect of the 2 factors. In the ANOVA, the outcome variable is the expert evaluation score. The follow-up survey data will be analyzed using correlations to examine the relationship between participants’ self-rated creativity, the average time per hypothesis generation, the number of hypotheses generated, and the expert evaluation score. The SUS will be used to assess the usability of VIADS. Qualitative data from the SUS surveys will be used to guide revisions of VIADS after Study 1. Descriptive statistics will also be used to report the answers to other follow-up questions.

A *t* test will be conducted to determine whether the revised VIADS improves the performance of research hypothesis generation in Study 2.

## Results

### Overview

The study is a National Institutes of Health–funded R15 (Research Enhancement Award) project supported by the National Library of Medicine. We began collecting data in July 2021 via pilot studies, and here provide some preliminary results and summarize our early observations. The full results and analysis of the study will be shared in future publications when we complete the study.

### Instruments

Based on a literature review, metrics were developed to assess research hypotheses [1,2,4,6-9,39]. Most of the dimensions used to evaluate clinical research hypotheses include clinical and scientific *validity*; *significance* (regarding the target population, cost, and future impact); *novelty* (regarding new knowledge, impact on practice, and new methodology); *clinical relevance* (regarding medical knowledge, clinical practice, and policies); *potential benefits and risks*; *ethicality*; *feasibility* (regarding cost, time, and the scope of the work); *testability*; *clarity* (regarding purpose, focused groups, variables, and their relationships); and *researcher interest level* (ie, willingness to pursue).

Multiple items were used to measure the quality for each dimension mentioned above. For each item, a 5-point Likert scale (1=strongly disagree, 5=strongly agree) was used for measurement. After internal consensus, we conducted external consensus and sought feedback from the external expert panel via an online survey [44]. The metrics are revised continuously by incorporating feedback. The expert panel will use our online survey [45] to evaluate research hypotheses generated by research participants during the study sessions.

We have developed the initial study scripts for Study 1 and have revised them after the pilot study sessions (Study 0). We have developed the screening survey for the recruitment process. The follow-up survey is administered after each study session, regardless of the group. The standard SUS survey [46,47] has been modified to add one more option in order to allow users to elaborate on what caused any dissatisfaction during the usability study.

### Recruitment

Currently, we are recruiting all levels of participants, including inexperienced clinical researchers, experienced clinical researchers, and a panel of clinical research experts. Recruitment began in July 2021 with pilot study participants. To participate, anyone involved in clinical research can share their contact email address by filling out the screening survey [48]. So far, we have completed 16 study sessions with inexperienced clinical researchers who have either used or not used VIADS in Study 1.

### Study 1

For this study, we are using data from the National Ambulatory Medical Care Survey (NAMCS) conducted by the Centers for Disease Control and Prevention [36]. The NAMCS is a publicly accessible data set of survey results related to clinical encounters in ambulatory settings. We processed raw NAMCS data (ICD9 codes and accumulated frequencies) from 2005 and 2015 to prepare the needed data sets for VIADS based on our requirements.

The experience level of the clinical researchers was determined by predetermined criteria. To determine which group a participant joins (inexperienced [groups 2 and 4] or experienced [groups 1 and 3] clinical researchers), we used the R statistical software package (blockrand [49]) to implement block randomization. The random blocks range from 2 to 6 participants.

### Initial Observations

We have noticed that both forward [27] and backward reasoning had been used by participants during the study sessions. In addition, some participants did not start from data or a hypothesis. Instead, the reasoning started from the participant’s focused (and often familiar) area of knowledge related to several ICD9 codes in the focus area being examined. The research hypotheses were then developed after examining the data on the focused area.

Many participants did not use any advanced analysis during the study sessions. However, they did use their prior experience and knowledge to generate research hypotheses based on the frequency rank of the provided data sets and by comparing the 2 years of data (ie, 2005 vs 2015).

Noticeably, VIADS can answer more complicated questions both systematically and more rapidly. However, we noticed that the training session required to enable use of VIADS increased participants’ cognitive load. Cognitive load refers to the amount of working memory resources required during the task of thinking and reasoning. Without a comprehensive analysis, we cannot yet draw further conclusions about the potential effects of this cognitive load.

## Discussion

### Significance of the Study

A critical first step in the life cycle of any scientific research study is formulating a valid and significant research question, which can usually be divided into several scientific hypotheses. This process is often challenging and time-consuming [1,3,38,50]. Currently, there is limited practical guidance regarding generating research questions [38] beyond emphasizing that it requires long-term experience, observation, discussion, and exploration of the literature. A scientific hypothesis–generation process will eventually help to formulate relevant research questions. Our study aims to decipher the process of scientific hypothesis generation and determine whether a secondary data analytics tool can facilitate the process in a clinical research context. When combined with clinical researchers’ experiences and observations, such tools can be anticipated to facilitate scientific hypothesis generation. This facilitation will improve the efficiency and accuracy of scientific hypothesis testing, formulating research questions, and conducting clinical research in general. We also anticipate that an explicit description of the scientific hypothesis–generation process with secondary data analysis may provide more feasible guidance for clinical research design newcomers (eg, medical students and new clinical investigators). However, we have not completed all study sessions, so we cannot yet analyze the collected data in order to draw meaningful conclusions.

### Interpretation of the Study and Results

Participants have been observed to use analogical reasoning [51] both consciously and subconsciously; meanwhile, some participants verbally expressed that they avoided analogical reasoning intentionally to be more creative during the study sessions. The participants intentionally did not use the same pattern of statements for all the topics supported by the data sets. The way we organized the data sets seems to promote the participants to think systematically when using the data sets. For instance, the use frequencies of ICD9 codes were sorted from high to low in each data set. However, what would constitute the perfect balance between systematic structure and randomness during scientific hypothesis generation is unknown. Intuitively, both systematic reviews and random connections should be critical in generating novel ideas in general, regardless of academic settings or industrial environments. Concrete evidence is needed to draw any conclusions about the relationships between the 2 during scientific hypothesis generation with certainty. Additionally, the current version of VIADS can only accept coded data using ICD9-CM, ICD10-CM, and MeSH. This inevitably limits the types of hypotheses VIADS can generate. We also recognize that other more broadly used hierarchical terminologies, such as SNOMED CT, RxNorm, and LOINC, could provide additional valuable information related to more comprehensive aspects of clinical care. However, our current version of VIADS cannot use such information at this time.

Analyzing the research hypothesis–generation process may include several initial cognitive components. These components can consist of searching for, obtaining, compiling, and processing evidence; seeking help to analyze data; developing inferences using obtained evidence and prior knowledge; searching for external evidence, including literature or prior notes; seeking connections between evidence and problems; considering feasibility, testability, ethicality, and clarity; drawing conclusions; formulating draft research hypotheses; and polishing draft research hypotheses [1-3,8,39,52]. These initial components will be used to code the recorded think-aloud sessions to compare differences among groups.

We recognize that research hypothesis generation and the long refining and improving process matter most during the study sessions. Without technologies to capture what occurs cognitively during the research hypothesis–generation process, we may not be able to answer fundamental questions regarding the mechanisms of scientific hypothesis generation.

Establishing the evaluation metrics to assess research hypotheses is the first step and the critical foundation of the overall study. The evaluation metrics used will determine the quality measurements of the research hypotheses generated by study participants during the study sessions.

Research hypothesis evaluation is subjective, but metrics can help standardize the process to some extent. Although the metrics may not guarantee a precise or perfectly objective evaluation of each research hypothesis, such metrics provide a consistent instrument for this highly sophisticated cognitive process. We anticipate that a consistent instrument will help to standardize the expert panel’s evaluations. Additionally, objective measures, such as the number of research hypotheses generated by the study participants and the average time each participant spends generating each research hypothesis, will be used in the study. The expert panel is therefore expected to provide more consistent research hypothesis evaluations with the combined metrics and objective measures.

Although developing metrics appears linear, as presented in Figure 2, the process itself is highly iterative. No revision occurs only once, and when we reflect on the first 3 stages of development, one observes that major revisions during the first 3 stages involve separating questions in the survey and refining the options for the questions. These steps reduce ambiguity.

### Challenges

Many challenges have been encountered while conducting the research hypothesis–generation study sessions. These include the following:

1. What can be considered a research hypothesis? What will not be considered a research hypothesis? The response will determine which research hypotheses will be evaluated by the panel of clinical research experts.
2. How should the research hypothesis be measured accurately? Although we developed workable metrics, the metrics are not yet perfect.
3. How can we accurately capture thinking, reasoning, and networking processes during the research hypothesis–generation process? Currently, we use the think-aloud method. Although think-aloud protocols can capture valuable information about the thinking process, we recognize that not all processes can be articulated during the experiments, and not everyone can articulate their processes accurately or effectively.
4. What happens when a clinical researcher examines and analyzes a data set and generates a research hypothesis subconsciously?
5. How can we capture the roles of the external environment, internal cognitive capacity, existing knowledge base of the participant, and interactions between the individual and the external world in these dynamic processes?

When faced with challenges, we see opportunities for researchers to further explore and identify a clearer picture of research hypothesis generation in clinical research. We believe that the most pressing target is developing new technologies in order to capture what clinical researchers do and think when generating research hypotheses from a data set. Such technologies can promote breakthroughs in cognition, psychology, computer science, artificial intelligence, neurology, and clinical research in general. In clinical research, such technologies can help empower clinical researchers to conduct their tasks more efficiently and effectively.

### Lessons Learned

We learned some important lessons while designing and conducting this study. The first lesson involved balancing the study design (straightforward or complicated) and conducting the study (feasibility). During the design stage, we were concerned that the 2×2 study design was too simple, even though we know it does not negatively impact the value of the research. We simply considered experience levels and whether the participants used VIADS in a very complicated cognitive process. However, even for such a straightforward design, only 1 experienced clinical researcher has volunteered so far. Thus, we will first focus on inexperienced clinical researchers. Even for study sessions involving inexperienced clinical researchers, considerable time is needed to determine strategies for coding and analyzing the raw data. In order to design a complicated experiment that answers more complex questions, we must consider balancing practical workload, recruitment reality, expected timeline, and researchers’ desire to pursue a complex research question.

Recruitment is always challenging. Many of our panel invitations to clinical research experts either received no response or were rejected, which significantly delayed the study timeline, in addition to the effects of the COVID-19 pandemic. Furthermore, the IRB approval process was time-consuming, delaying our study when we needed to revise study documents. Therefore, the study timeline includes the IRB initial review and rereview cycles.

### Future Work

The first step of a future direction for this project is to explore the feasibility of formulating research questions based on research hypotheses. In this project, we are looking for ways to improve the efficiency of generating research hypotheses. The next step will be to explore whether we can enhance the efficiency of formulating research questions.

A possible direction for future work is to develop tools to facilitate scientific hypothesis generation guided by secondary data analysis. We may explore automating the process or incorporating all positive attributes in order to guide the process better and improve efficiency and quality.

At the end of our experiments, we asked clinical researchers what facilitates their scientific hypothesis–generation process the most. Several of their responses included repeatedly reading academic literature and discussing with colleagues. We believe intelligent tools can undoubtedly improve both aspects of scientific hypothesis generation, namely, summarizing new publications of the chosen topic areas and providing conversational support to clinical researchers. This would be a natural extension of our studies.

An additional possible direction is to expand the terminologies that can be used by VIADS, for example, the addition of RxNorm, LOINC, and SNOMED CT can be considered in the future.

## Appendix

Multimedia Appendix 1 Follow-up survey at the end of the hypothesis-generation tasks.

Multimedia Appendix 2 VIADS System Usability Scale survey and utility questionnaire.

Multimedia Appendix 3 List of items to consider during hypothesis generation.

## Abbreviations

ICD: International Classification of Diseases
ICD9-CM: International Classification of Diseases, 9th Revision-Clinical Modification
ICD10-CM: International Classification of Diseases, 10th Revision-Clinical Modification
IRB: Institutional Review Board
LOINC: Logical Observation Identifiers Names and Codes
MeSH: Medical Subject Headings
NAMCS: National Ambulatory Medical Care Survey
SNOMED-CT: Systematized Nomenclature of Medicine-Clinical Terms
SUS: System Usability Scale

## Data Availability

A request for aggregated and anonymized transcription data can be made to the corresponding author, and the final decision on data release will be made on a case-by-case basis, as appropriate. The complete analysis and the results will be published in future manuscripts.
